# Non-suicidal self-injury in adolescence: a validation of the Chinese version of the Inventory of Statements About Self-Injury in student populations

**DOI:** 10.3389/fpsyt.2025.1510681

**Published:** 2025-02-27

**Authors:** Xinhe Tian, Hebin Huang, Weicong Lu, Ruoxi Zhang, Runhua Wang, Xiaoyue Li, Danping Li, Yanling Gao, Shiyun Wu, Guiyun Xu, Robin Shao, Kangguang Lin

**Affiliations:** ^1^ Department of Affective Disorder, The Affiliated Brain Hospital, Guangzhou Medical University, Guangzhou, Guangdong, China; ^2^ Key Laboratory of Neurogenetics and Channelopathies of Guangdong Province and the Ministry of Education of China, Guangzhou Medical University, Guangzhou, Guangdong, China; ^3^ State Key Laboratory of Brain and Cognitive Sciences, Department of Psychology, University of Hong Kong, Hong Kong, Hong Kong SAR, China; ^4^ School of Health and Life Sciences, University of Health and Rehabilitation Sciences, Qingdao, Shandong, China; ^5^ Department of Neurology, Lecong Hospital of Shunde, Foshan, Guangdong, China

**Keywords:** non-suicidal self-injury, Chinese, measurement, validation, self-injurious behavior

## Abstract

**Background:**

The Inventory of Statements About Self-Injury (ISAS) is a widely utilized scale for evaluating the 13 potential functions that motivate non-suicidal self-injury(NSSI) behaviors. However, its validation for assessing such motivation in a Chinese context is still lacking.

**Aims:**

The main objective was to access the validation of ISAS as an instrument in Chinese young population.

**Method:**

A total of 1,106 completed online self-report questionnaires were collected, with 167 reporting a history NSSI. The age range of these individuals was 12 to 24 years old, comprising 74 female and 93 male participants. The factor structure and construct validity were calculated using exploratory factor analysis (EFA) and confirmatory factor analysis (CFA). The correlations of the Patient Health Questionnaire-9 (PHQ-9), the Generalized Anxiety Disorder-7 (GAD-7), the Brief Self-Control Scale (BSCS), the Self-Rating Idea of Suicide Scale (SIOSS), and the Chinese version of the ISAS were tested using bivariate correlation analyses.

**Results:**

The internal consistencies of the Chinese version of the ISAS were good to excellent, with 0.788- 0.950 and 0.80-0.949 in the sports group and the junior high school group, respectively. EFA and CFA exhibited a good two-factor structure model (NFI = 0.942, CFI = 0.974, IFI = 0.974, RMSEA = 0.068, SRMR = 0.043, CMIN/DF = 1.762). Moreover, the scores of the functions of the ISAS were correlated with depression (r=0.208, p<0.01), anxiety (r=0.223, p<0.01), suicidal ideation (r=0.322, p<0.01), and low self-control (r=-0.230, p<0.01).

**Conclusion:**

This study validates the Chinese ISAS as a reliable NSSI measure, identifies a two-factor structure, and aims to inform targeted interventions and future research on self-injury behaviors among Chinese adolescents.

## Introduction

Nonsuicidal self-injury (NSSI) refers to a range of specific self-injurious behaviors (SIB) such as intentional injury of body tissue without subjective suicidal intent (1, 2). Similar terminology such as deliberate self-harm, self-destruction, and deliberate self-cutting has also been used in existing literature. Behaviors considered to be self-injurious are varied, with the most frequently mentioned self-injury behaviors including cutting, scratching, hitting, hair-pulling, and biting. Considering the significant impact of suicide and mental disorders on global mortality, causing approximately 14.3% or 8 million deaths annually (3), it is crucial to note that previous studies have identified NSSI as a prevalent risk factor for both suicidal behaviors (4, 5) and various severe psychopathological conditions such as disordered eating behaviors (6), borderline personality disorder, and depression (7). The escalating prevalence of NSSI behaviors worldwide (8) indicates that mental health challenges and high-risk behaviors are not isolated to specific countries but rather a pervasive issue on a global scale.

### Cultural context and limitations of existing measures

While existing self-injury assessment tools have been widely used and validated in various cultural contexts, their application in China presents unique challenges. The cultural context in which NSSI occurs in China is significantly different from that in Western countries. Studies indicate that the initiation of self-injurious behaviors typically commences during the adolescent stage, with a lifetime prevalence rate being approximately 17.1% to 38.6% in European countries (9) and 11.5% to 33.8% in developing countries (10). In China, the occurrence of NSSI behaviors among middle school students has been reported to range from 0.06% to 22.7% (11, 12), with a higher prevalence of 29.2% observed in rural areas (13). Consistent with the meta-analyses of self-injurious behavior in global adolescent populations (14), Chinese adolescents showed significance for adverse life events, negative coping style, problematic internet use, sleep disturbance, traumatic experiences, problematic parent-child relationship, mental health problems. However, it also emphasized some specific circumstances (15): the collectivist nature of Chinese society, where individual behavior is often influenced by group norms and expectations, can affect how individuals express and understand their self-injurious behaviors (16). Additionally, socio-cultural factors such as China’s one-child policy, the phenomenon of left-behind children (17), and the widely used internet culture (18) have been identified as specific circumstances influencing the occurrence of NSSI. These factors highlight the need for a culturally appropriate assessment tool that can accurately capture the unique motivations and experiences of individuals engaging in NSSI in China.

Despite the availability of a variety of assessment tools for NSSI, such as the Inventory of Statements About Self-Injury (ISAS), the Self-Injury Questionnaire-Treatment Related (SIQ-TR), the Self-Injurious Thoughts and Behavior Interview (SITBI), the Functional Assessment of Self-Mutilation (FASM), the Non-Suicidal Self-Injury Assessment Tool (NSSI-AT), and the Ottawa Self-Injury Questionnaire, some of these tools still have limitations in practical application. They mainly focus on assessing the frequency and severity of NSSI behaviors but fall short in evaluating the underlying motivation and psychological mechanisms (19, 20). This limits our comprehensive understanding of the complexity of NSSI and also impacts the effectiveness of interventions (21).

### Need for a culturally appropriate assessment tool

Significant progress has been made in the research NSSI, and a variety of measurement tools have been developed. However, it is essential to carefully examine the potential drawbacks of these tools, such as the heterogeneity among different scales (22). This highlights the necessity of assessing the cross-cultural consistency of these scales. For example, the ISAS, which has been validated in multiple countries (23–26), may not fully capture the cultural nuances that influence NSSI in the Chinese context. Some terms and concepts used in Western scales may not resonate with Chinese participants, potentially leading to misunderstandings or misinterpretations. Moreover, the use of measurement tools designed for different cultures can introduce measurement bias, thereby affecting the validity and reliability of the results. Therefore, it is crucial to develop a Chinese version of the ISAS. By incorporating language appropriate to the Chinese cultural context and ensuring that the scale is relevant and sensitive to the Chinese situation, a culturally adapted version of the ISAS will be able to effectively address these issues.

### Importance of understanding NSSI motivations

Exploring the motivations behind NSSI behaviors has been recognized as a valuable approach to comprehend the underlying causes and subsequently develop targeted interventions and preventive measures. Previous studies have repeatedly examined the seven functions of NSSI (i.e., affect-regulation, anti-dissociation, anti-suicide, interpersonal boundaries, interpersonal-influence, self-punishment, and sensation-seeking) (1). Among the scales that include the assessment of motivations for self-injury, the ISAS and FASM are the most widely used (22). The FASM (27) identifies four functions of self-harm (i.e., auto matic-positive reinforcement, automatic negative reinforcement, social-positive reinforcement, social negative reinforcement) (28) by asking participants about 22 potential reasons (e.g. to avoid school, work, or other activities) of self-harm, while the ISAS, based on relevant self-harm theories (1, 29), encompasses two domains, namely interpersonal functions and intrapersonal functions, summarizing 13 functions of self-harm (e.g., interpersonal influence and affect regulation). Klonsky and Glenn validated the ISAS scale through a sample of 235 young adult college students (29) and later assessed the scale’s one-year test-retest reliability (30). Understanding the motivations behind NSSI will not only enhance our comprehension of the psychometric properties underlying NSSI engagement but also enable the identification of individuals who are more vulnerable to such behaviors in a globally comparable manner. This, in turn, will facilitate the development and implementation of more effective support and intervention strategies.

### Purpose of the present study

Although there have been many validation studies of self-harm related scales(such as FASM) in China (31, 32), studies on the validation of the Chinese version of the ISAS remain limited (33). To provide a detailed understanding of the subjective functions of NSSI and offer a new way of investigating NSSI motivation in the Chinese cultural context, it is important to verify the reliability and validity of the ISAS in Chinese. The current study had two goals: the first was to investigate the factor structure and internal consistency of the Chinese version of the Inventory of Statements about Self-Injury (ISAS), and the second was to evaluate a specific demographic’s behavior and their motivation for NSSI behaviors. It was hypothesized that the Chinese version of the ISAS would establish a well two-factor structure providing adequate psychometric features.

## Methods

### Sample

The study cohort comprised Chinese adolescents aged 12 to 24 years, who were enrolled from two schools participating in distinct research projects during the period from October to December 2021. The majority of participants were from a secondary school in Huaiji, a county in Guangzhou. In total, 706 first year junior high school students completed the questionnaire online, among who 182 students reported NSSI history. The remaining participants were recruited from Guangzhou Sport University and completed the questionnaire on Redcap (a questionnaire distribution platform). In total, 464 completed questionnaires were received from the Sport University students, among who 49 students reported NSSI history.

Participants who reported NSSI history were excluded if their responses were inconsistent or obviously irrational. 14 university and 44 junior high school students were excluded because they had not done any self-harm behavior but reported at least one way of NSSI behavior. Six junior high school students were excluded because their responses were irrational (i.e. times of self-harm behavior >1000 or irrelevant answers with NSSI history).

After excluded these participants, a total of 1,106 questionnaires were deemed eligible (450 university students and 656 adolescents from the junior high school student group). A total of 167 students, consisting of 132 high school students and 35 university students, reported engaging in at least one incident of NSSI during their lifetime. This final sample of 167 NSSI students was used in the validation study. The age data for some university students were missing (N=4), but these gaps were filled using the mean substitution method.

### Instrument

Inventory of Statements about Self-Injury (ISAS). The scale is made up of two parts. The first part of the ISAS assesses the lifetime frequency of 12 “intentional” (i.e., on purpose) and “non-suicidal” self-injury behavior types and one blank which can be filled out by participants in the event that they had engaged in another self-harm behavior type not already mentioned. The specific assessed behaviors include: cutting, severe scratching, biting, banging or hitting self, burning, interfering with wound healing (e.g. picking scabs), carving, rubbing skin against rough surface, pinching, sticking self with needles, pulling hair, and swallowing dangerous substances. Considering that it can be difficult for students to recall the specific number of self-injury behaviors and to facilitate statistical analysis, we defined the lifetime frequency of self-injurious behaviors according to methods used in previous studies (34). The frequency responses are divided into a five-point Likert scale: 1 (none), 2 (only once), 3 (2-10 times), 4 (11-50 times), and 5 (more than 50 times). The second part of the ISAS comprises 39 items assessing 13 potential functions of NSSI, however the respondent is only asked to complete this section if they have previously reported one or more NSSI behaviors. Each item is rated as 0 (not relevant), 1 (somewhat relevant), or 2 (very relevant) to represent their feelings regarding why they engaged in self-harm. Thus, the score for each of the 13 potential functions in ISAS is determined by adding up the scores of three specific items, resulting in a score ranging from 0 to 6. Each item starts with the stem “when I harm myself I am …”, and includes sample items such as “calming myself down” (affect regulation), “creating a boundary between myself and others” (interpersonal boundaries), “punishing myself” (self-punishment), “giving myself a way to care for myself (by attending to the wound)” (self-care), “causing pain so I will stop feeling numb” (anti-dissociation), “avoiding the impulse to attempt suicide” (anti-suicide), “doing something to generate excitement or exhilaration” (sensation-seeking), “bonding with peers” (peer-bonding), “letting others know the extent of my emotional pain” (interpersonal influence), “seeing if I can stand the pain” (toughness), “creating a physical sign that I feel awful” (marking distress), “getting back at someone” (revenge), “ensuring that I am self-sufficient” (autonomy).

Patient Health Questionnaire-9 (PHQ-9). The PHQ-9 is a validated depression rating scale which assesses symptoms over the preceding two weeks (35), and is frequently used in both community and clinical samples. It is useful as a quick screening and monitoring tool due to its short form and easy diagnosis. The PHQ-9 contain nine items, each rated from 0 (not at all) to 3 (nearly every day). The Chinese version of the PHQ-9 has demonstrated good reliability, with a Cronbach’s α of 0.86 (36).

Generalized Anxiety Disorder-7 (GAD-7). Because anxiety is one of the common risk factors of non-suicidal self-injury (4), the GAD-7 is usually used as a tool to assess the anxiety of participants over the previous two weeks (37). The scale consists of seven items which are graded from 0 (not at all) to 3 (nearly every day). The total score ranges from 0 to 21. The Chinese version of the GAD-7 has demonstrated good reliability, with Cronbach’s α ranging from 0.93 to 0.95 (38).

Brief Self-Control Scale (BSCS). Self-control is defined as the ability to maintain one’s attention focused and overcome dominant responses in order to achieve a long-term goal (39). The BSCS is a seven-item short-form self-report inventory which assesses self-control using two domains: self-discipline and impulse control. The scale uses a five-point Likert scale ranging from 1 (not at all) to 5 (very much). A higher score indicates greater respondent self-esteem. The Chinese version of the BSCS demonstrates a Cronbach’s α of 0.83, which reflects acceptable levels of reliability and validity (40).

Self-Rating Idea of Suicide Scale (SIOSS). The SIOSS is a 26-item scale developed to measure Chinese respondents’ levels of suicidal ideation. Responses are scored using a two-point scale, where 0 = Yes and 1 = No. The measure has four subscales, despair (12 items), optimism (5 items), sleep (4 items), and hiding (5 items). The total suicidal ideation score is the sum of the first three subscales, and the total can range from 0 to 21 (Cronbach’s α = 0.79) (41). The “Hiding” subscale is considered to be a way to improve answers’ reliability and makes adjustments in consideration of the feeling of discomfort respondents feel when asked the suicide ideation items. Generally, a total score of 12 or more is considered to be presence of suicidal ideation.

### Design

To create the Chinese version of the ISAS we adopted a back-translation approach (42). The forward translation (from English to Chinese) was carried out by the first author (XT), who is a graduate student in psychiatry with a focus on research related to self-injurious behaviors. This ensured a profound comprehension of the original scale’s content and context. Subsequently, the backward translation (from Chinese to English) was performed by another graduate student in psychiatry (HH), who is proficient in both English and Chinese and has a solid research background in mental health. Both of these steps were supervised by the senior author (KL), who has extensive experience in scale development and validation. Thereafter, a panel of experts, including clinicians and researchers with expertise in psychiatry and psychometrics (such as WL, RZ, RW, etc.), compared and reconciled the translations to ensure the accuracy and cultural appropriateness of the translated items.

The questionnaire was then distributed to the participants after they received and completed the consent form which explained the purpose of the study. All participants returned the signed informed consent form, indicating their voluntary participation and understanding of the study’s requirements. For minor participants, the informed consent form was signed by their parents.

This study was voluntary and participants were free to withdraw at any point. The survey anonymized participants’ personal information, so the students’ identities cannot be determined, and the information they provided also cannot be leaked out to their peers or others. The questionnaire was distributed online, to further ensure privacy and data collection convenience. After completing the research questionnaire, students who felt disturbed due to the study’s focus or who required further emotional support were referred to the school’s psychology teacher as a first step, and then if they also reported high-risk thoughts or behaviors which had happened before, feedback and advice was provided to the student’s parents as to whether the student may require further clinical diagnosis or treatment.

Data were analyzed using SPSS 26 for Windows. Descriptive analyses (mean [M], standard deviation [SD], and frequencies) were used for the sample description and quantitative items of the ISAS.

Cronbach’s alpha (α) was used in SPSS to evaluate the internal consistency reliability of the questionnaire. It is generally recognized that, under the same conditions, a greater number of items typically leads to a higher Cronbach’s alpha value.

Validity analysis used exploratory factor analysis (EFA) and confirmatory factor analysis (CFA) to analyze the discriminant validity and the structure of the questionnaire.

Exploratory factor analysis (EFA) was calculated using SPSS and R with principal axis factoring and Varimax rotation to evaluate the factor structure of ISAS Section 2. The number of factors for the scale was determined using eigenvalues(>1), cumulative variance contribution rates(60%-70%), scree plots, parallel analysis (PA) (43), the Hull method and the assessment of the closeness of fit to unidimensionality (44).

The factor structure of ISAS Section 2 was also evaluated using Confirmatory Factor Analysis (CFA) in AMOS 28.0 for Windows. The method of maximum likelihood estimation was used, and a combination of various fit indices, including the chi-square test, the root mean square error of approximation (RMSEA), the standardized root mean squared residual (SRMR), the normed fit index (NFI), the comparative fit index (CFI), and the incremental fit index (IFI), were utilized to comprehensively assess the goodness-of-fit of the model to the data. The SRMR is a measure of the discrepancy between the observed data and the model-predicted data. A smaller SRMR value indicates a better model fit. Typically, an SRMR value less than 0.08 is considered to indicate a good fit (45). RMSEA is an important fit statistic, wherein a value of less than 0.05 indicates a good fit, nearly 0.08 indicates that reasonable errors exist, from 0.08 to 0.10 indicates a mediocre fit, and greater than 0.10 indicates a poor fit (46). Besides, a chi-square minimum/degree of freedom (CMIN/DF) value of less than 3.0 indicates an acceptable fit (47, 48).

The function subscales of the ISAS are treated as observed variables and are depicted as rectangles in the model. To examine whether the ISAS CFA model differs between genders and age groups, we conducted a multi-group simultaneous analysis. A critical ratio with an absolute value exceeding 1.96 was considered to indicate a significant difference, when the significance level was set at 0.05.

Bivariate correlation analyses were used to examine whether the ISAS and its subscales correlated with other variables in the expected directions, which helped evaluate the measure’s criterion related validity. In the present study, only students who reported a history of NSSI were included in the subsequent statistical analyses.

## Results

### Demographic characteristics

Of the two different groups of NSSI participants, university students account for 21.0%. 48.6% of the university students were male(N=, and 42.4% of the junior high school students were female. The university group participants were 18 to 24 years of age (M = 20.32, SD = 1.38), and the junior high school students were 12 to 16 years of age (M = 12.90, SD = 0.537).

### Section 1 of the ISAS (ISAS Behavioral Scales)

Cronbach’s alpha was used to analyze the internal consistency of the first section of the ISAS. The overall data are shown in **Table 1**. For the university group (**Supplementary Table S1a**) and the first year of junior high school group (**Supplementary Table S1b**) the coefficient alphas were 0.788 and 0.80, respectively. The overall standardized reliability coefficient in both groups were acceptable (0.799).

**Table 1:** The internal consistency of the first section of the ISAS.

ISAS behavior	Both groups			
	*M* (*SD*)	Sample percentage (%)	Correlations with the overall score	Cronbach's alpha if item deleted
Cutting	1.68(0.95)	40.1	0.565**	0.773
Severe scratching	1.44(0.82)	26.3	0.703**	0.756
Biting	1.53(0.84)	32.3	0.687**	0.757
Banging or hitting self	1.83(1.01)	45.5	0.642**	0.764
Burning	1.18(0.53)	12	0.451**	0.780
Interfering with wound healing (e.g., picking scabs)	2.14(1.31)	50.9	0.577**	0.784
Carving	1.65(1.00)	36.5	0.577**	0.772
Rubbing skin against rough surface	1.46(0.87)	26.9	0.630**	0.764
Pinching	1.61(0.99)	32.9	0.539**	0.777
Sticking self with needles	1.14(0.47)	9.6	0.497**	0.778
Pulling hair	1.47(0.96)	24.6	0.535**	0.776
Swallowing dangerous substances	1.03(0.17)	3	0.246**	0.791
Other	1.05(0.27)	3.6	0.203**	0.791

Cronbach’s alpha Based on standardized items: 0.799.

**Correlation is significant at the 0.01 level (two-tailed).

In the university group, “Swallowing dangerous substances” was not selected by any student, but more than half of the students reported “Interfering with wound healing (e.g., picking scabs)” and “Banging or hitting self” as ways they had hurt themselves before (57.1% and 54.3%, respectively), which means that some students chose more than one way to harm themselves. According to the reliability coefficient of the deleted item, after deleting the “Pulling hair” item the coefficient alphas were greater than before the item deletion. The correlation with the overall value after deletion was 0.217 (less than the judgment criterion 0.3), so after re-analysis we considered deleting the items. The reliability analysis after deletion found that α = 0.809, with all other 11 items included. However, considering that the imperfect reliability of Section 1 could have been due to an insufficient sample size, and as the reliability value of the original version of the ISAS was acceptable, the first version was preserved. Other studies have found that there are some differences between males and females in terms of self-injury behavior choice. For the university group, females chose “cutting” (p = .007), “severe scratching” (p = .006), and “biting” (p = .014) as self-injury behaviors, all of which were significantly higher than the male rates.

“Interfering with wound healing (e.g., picking scabs)” was the most common form of self-injury in the junior high school student group (49.2%), as well as in the university group. “Cutting” and “banging or hitting self” followed (43.9% and 43.2%, respectively). Among junior high school students, the method of “Pinching” (p = .01) was found to be more frequently chosen by girls than boys.

### Section 2 of the ISAS (ISAS Functional Scales) - reliability analysis

The Cronbach’s alpha coefficients of the total items for the ISAS Section 2 was 0.950 in the university group and 0.949 in the junior high school group, indicating excellent internal consistency reliability. The whole section comprises two ISAS factors (i.e., interpersonal and intrapersonal) with 13 functions beneath these two factors. The Cronbach’s alpha coefficients can be further calculated by the scores of the 13 functions and by the affiliated two factors. The total Cronbach’s alpha coefficients calculated by the 13 functions was 0.923 in the university group (**Supplementary Table S2a**) and 0.93 in the junior high school group (**Supplementary Table S2b**), a little bit lower than that of all items. The overall standardized reliability coefficient in both groups was 0.929 (**Table 2**). The Cronbach’s alpha coefficients for the interpersonal and intrapersonal factors, computed using the 13 functions, demonstrated high internal consistency. In the university group, these coefficients were 0.946 and 0.784 for the interpersonal and intrapersonal factors, respectively. Similarly, in the junior high school group, the corresponding coefficients were 0.924 and 0.862.

**Table 2:** Means and standard deviations and the internal consistency for the functional subscale of ISAS.

Reliability Statistics for both groups				
	*M* (*SD*)	Sample percentage (%)	Correlations with the overall score	Cronbach's alpha if item deleted
Interpersonal boundaries	0.45 (0.88)	29.3	.765**	0.911
Self-care	0.77 (1.15)	43.7	.815**	0.908
Sensation seeking	0.40 (0.86)	22.8	.748**	0.912
Peer bonding	0.44 (1.08)	21.6	.732**	0.911
Interpersonal influence	0.50 (0.97)	26.9	.759**	0.911
Toughness	0.59 (1.11)	29.3	.773**	0.910
Revenge	0.23 (0.78)	12	.676**	0.914
Autonomy	0.52 (1.05)	25.7	.711**	0.912
Affect regulation	1.84 (1.57)	73.1	.635**	0.920
Self-punishment	1.26 (1.45)	58.1	.749**	0.912
Anti-dissociation	1.00 (1.27)	50.9	.792**	0.909
Anti-suicide	1.04 (1.57)	40.7	.642**	0.919
Marking distress	0.72 (1.21)	36.5	.715**	0.912

Cronbach’s alpha based on standardized items: 0.929.

**Correlation is significant at the 0.01 level (two-tailed).

Among university students, the most common reasons for engaging in NSSI behavior were the affect regulation function (82.9%) and self-punishment function (80%). Similarly, for junior high school students, the primary reasons for NSSI behaviors were also affect regulation function (70.5%) and self-punishment function (52.3%), mirroring the university group. Interestingly, across both groups, four students (one from the university group and the remaining from the junior high school group) reported the same alternative reason for their NSSI behavior, namely “feeling irritable”.

The correlation coefficient between the 13 functional factors and the total score was 0.380-0.823 in the university group and 0.632-0.832 in the junior high school group. All correlations between functions and overall score were statistically significant (P <.05), which means that even though deleting the “affect regulation” item in the university group resulted in greater coefficient alphas than with it included, reserving this significant correlational item was acceptable. The correlation coefficients between the two factors (interpersonal and intrapersonal) and the total score were 0.924 and 0.842, respectively, in the university group (p <.01). Similarly, the respective correlation coefficients were found to be 0.915 and 0.899 in the junior high school group (p <.01).

Otherwise, no differences between the sexes in functional factors were found in the sample of university students, but the presence of differences between the sexes was indeed observed in the sample of junior high school group. Compared to boys, young adolescent girls were more likely to do self-harm behaviors for affect regulation (p=0.048). However, boys were more prone to engage in self-injurious behaviors because of peer-bonding (p=0.01), toughness(p=0.028), revenge (p=0.003) and autonomy (p=0.008).

### Section 2 of the ISAS (ISAS Functional Scales) - validity analysis

The coefficient of the KMO test was 0.813 in the university group and 0.899 in the junior high school group. Both groups exhibited a significance level below 0.05 for the Bartlett’s test. Furthermore, the combined group KMO test coefficient was 0.913, indicating that the data is suitable for further factor analysis. Considering that good reliability and validity had already been tested for both of the groups, further EFA and CFA were used to examine the structure of the psychometric properties and the structure model of the ISAS functions using the datasets of the two groups together.

### Exploratory Factor Analysis (EFA)

**Figure 1:**
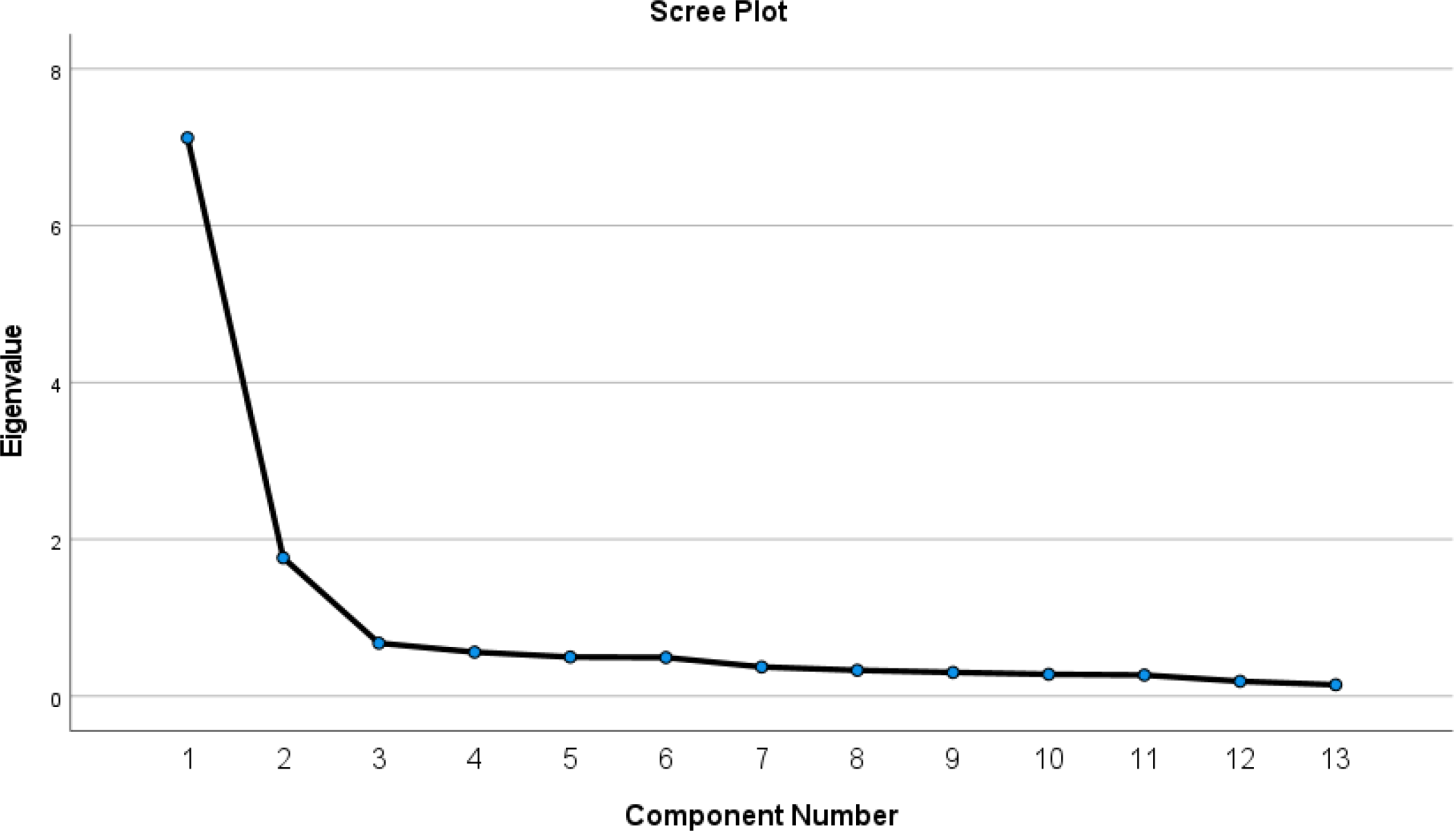
Scree plot in whole sample (n=167).

**Figure 2:**
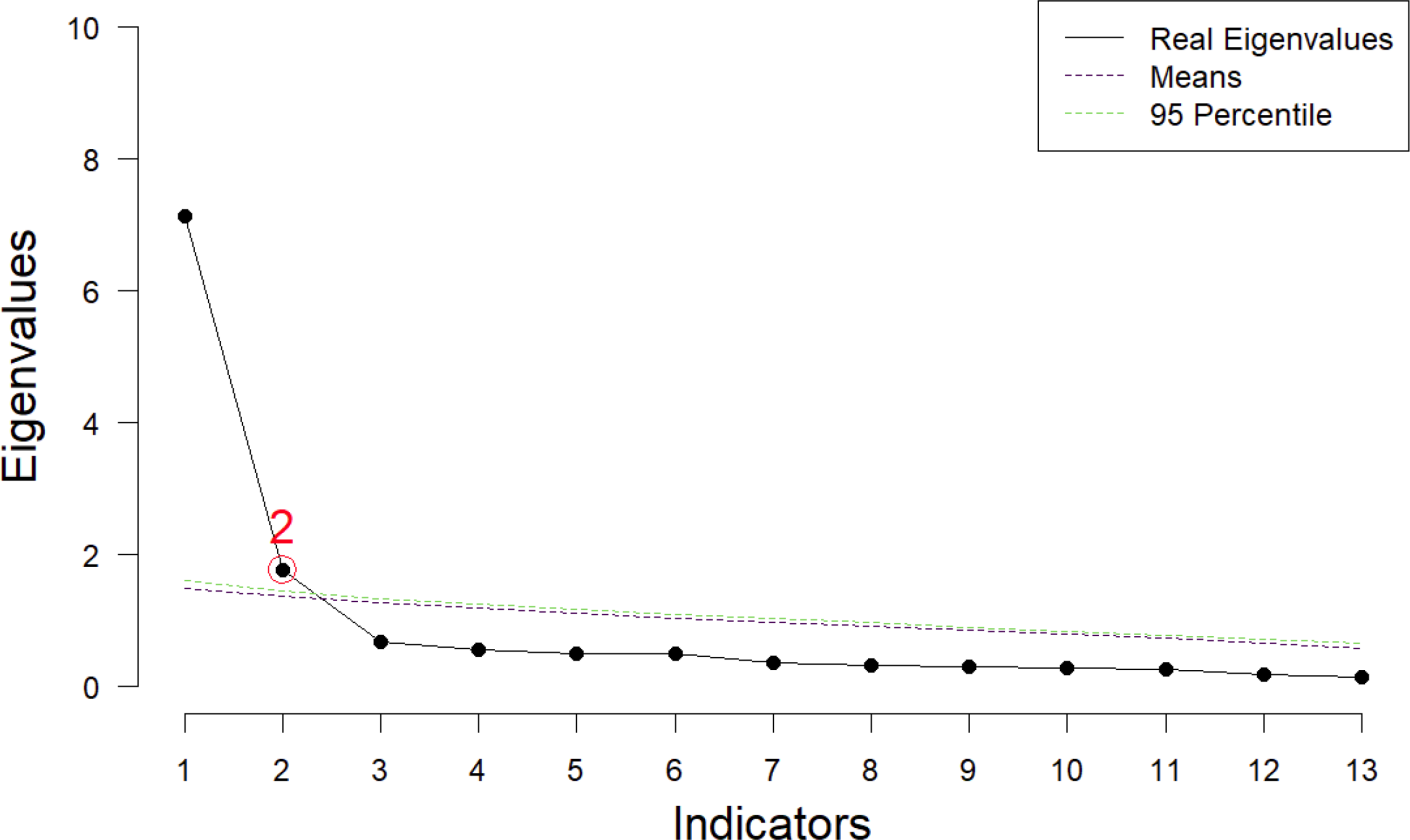
Real and random-data eigenvalues on parallel analysis.

To verify the psychometric properties of the ISAS Section 2, EFA was first conducted (see **Table 3**). The eigenvalues and scree plot (**Figure 1**) indicate an acceptable two-factor structure accounting for 68.346% of variance, which is consistent with the results of previous ISAS psychometrical research on NSSI functions (27). Factor 1 had an eigenvalue of 5.338 and Factor 2 had an eigenvalue of 3.547. Besides, PA based on principal component analysis of 1,000 random correlation matrices (43) and the Hull method (49) based on CFI and RMSEA both indicate that the recommended number of dimensions is 2 (**Figure 2**). Moreover, the results of the assessment of the closeness of fit to unidimensionality (44) indicate that the unidimensional congruence (UniCo) value is 0.918; a value greater than 0.95 would suggest that the data can be considered essentially unidimensional. The explained common variance (ECV) value is 0.604, which is below the recommended value of 0.85, confirming that the data cannot be regarded as essentially unidimensional. Finally, the mean of item residual absolute loadings (MIREAL) value is 2.01, which is much higher than 0.300, further supporting that the scale is more appropriately explained by a two-factor model.

**Table 3:** Standardized factor loadings for the two-factor model derived by EFA (N = 167).

Structure of the ISAS functions in Both Groups		
	Factor 1: Interpersonal	Factor 2: Intrapersonal
Interpersonal boundaries	**0.764**	0.318
Self-care	0.561	**0.611**
Sensation seeking	**0.767**	0.293
Peer-bonding	**0.899**	0.107
Interpersonal influence	**0.755**	0.31
Toughness	**0.75**	0.328
Revenge	**0.85**	0.082
Autonomy	**0.789**	0.191
Affect regulation	0.023	**0.894**
Self-punishment	0.246	**0.832**
Anti-dissociation/feeling-generation	0.422	**0.717**
Anti-suicide	0.191	**0.709**
Marking distress	0.539	0.471

Factor loadings greater than 0.6 are bolded.

The first factor seemed to include eight functions (i.e., interpersonal boundaries, interpersonal influence, peer-bonding, revenge, sensation seeking, toughness, autonomy, and marking distress), which represented interpersonal aspect. The second factor in both groups incorporated five functions (i.e., self-care, affect regulation, anti-dissociation, anti-suicide, self-punishment), representing intrapersonal aspect. No significant difference was found in the “marking distress” function between the two factors.

### Confirmatory Factor Analysis (CFA)

**Figure 3:**
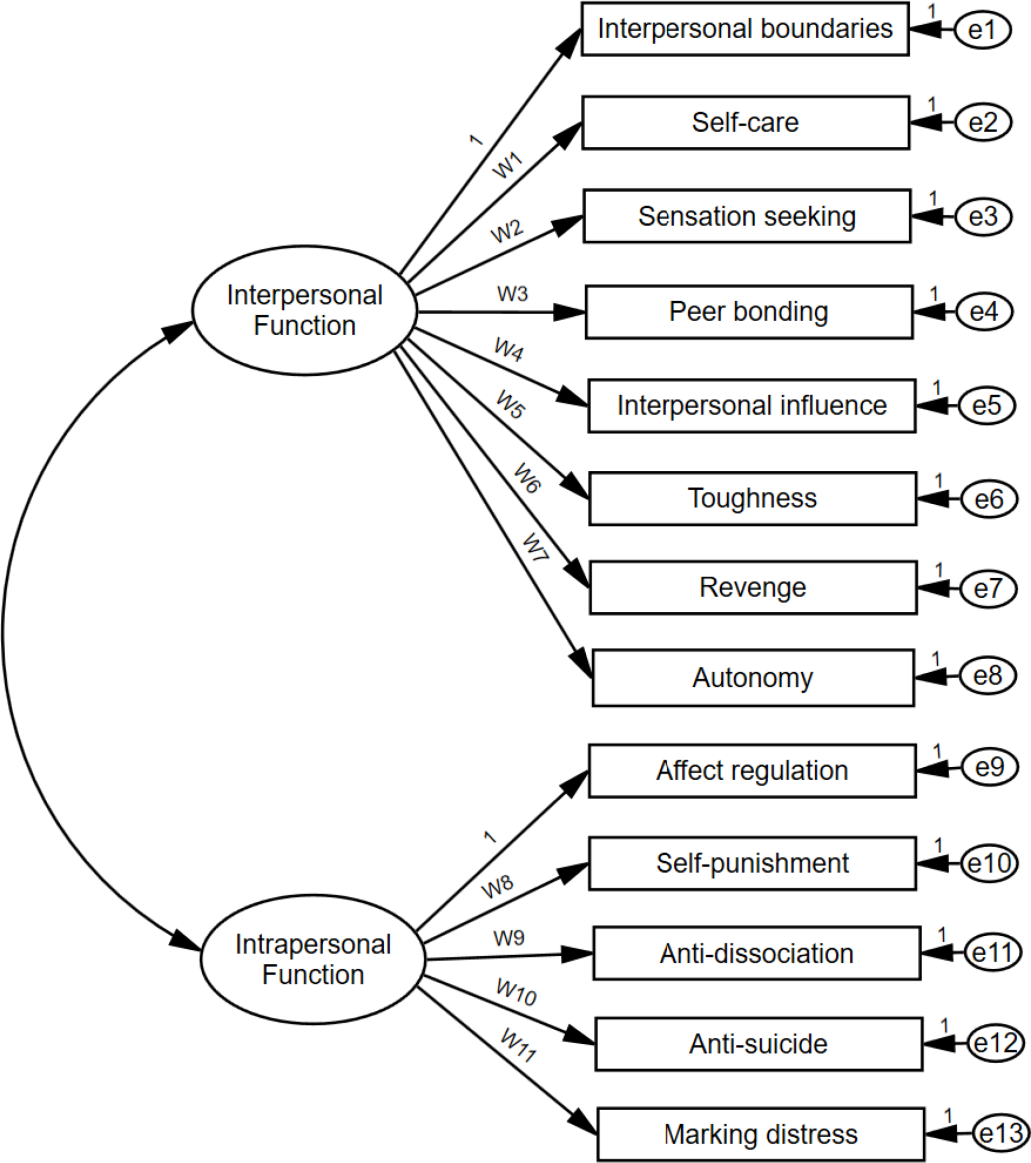
The two-factor model for the Chinese version of Inventory of Statements about Self-Injury (ISAS).

CFA of ISAS Section 2 was conducted using Amos 28 to explore whether NSSI functions demonstrate a two-factor structure in terms of construct validity, as defined by previous study (29), and to examine if the relationship between function subscales and factors aligns with the expected structure within Chinese cultural context. The current analysis developed a measurement model, mirroring the factor analysis results of the authors. This model included 13 observed variables and 2 latent variables, with factor loadings denoted by capital W (**Figure 3**).

The original CFA is a purification indicator measurement model, errors in observed variables were specified as independent from one another. The model results indicated a not satisfactory fit with the dataset. The five fit indices were not adequately acceptable (NFI = 0.833, CFI = 0.867, IFI = 0.869, RMSEA = 0.135, SRMR =0.097, CMIN/DF = 4.045). All these fit indices indicate a poor model fit. Based on the modification indices provided by the CFA results as well as supported by previous studies (1, 29), 13 correlated residuals between functions were established. It was seen that these pairs of variables, whose error variances were related, belonged to the observed variables within the same factors. Due to their semantic proximity, suggestions were made to consider them related to each other, which relaxed the assumption of independence without altering the theoretical structure. Based on the high factor loadings of “self-care” in the interpersonal factor revealed by the EFA results, a cross-factor correlation was established between the residuals of “self-care” and the residuals of specific intrapersonal factor observed variables. In conclusion, this new modified model was retested by CFA, showing a significant improvement of the results in terms of model fit. The fit indices indicated that the goodness-of-fit of this modified model was adequate (NFI = 0.942, CFI = 0.974, IFI = 0.974, RMSEA = 0.068, SRMR = 0.043, CMIN/DF = 1.762). The factor loadings of observed variables on all latent factors range from 0.46 to 0.85, with a median value of 0.75. The correlation value (0.74) between the two latent variables indicated that these two can be interpreted by one higher order latent variable, which can be considered to be a total ISAS function. The modified model for the ISAS is shown in **Supplementary Figure S1**.

To examine the potential effects of respondents’ gender and age on W1-11 in **Figure 3**, multi-group simultaneous analyses were conducted for each function subscale of ISAS. Most parameters did not show significant differences in terms of gender and age. The only difference between genders manifested in W4 (Interpersonal influence), while the difference in age was observed in W6 (Revenge). Furthermore, when setting the significance level at 0.01, the parameters of both gender groups do not exhibit significant differences. The differences in age primarily account for the larger variations in sample sizes. Considering that the main focus of the study is on the overall self-harm high-risk population (adolescents and early adulthood), the model remains unchanged in the end.

### Relationship to other clinical measures

Comparing the psychological variables between the two groups, there were only significant differences in self-control and intrapersonal factors (p <.05). Other than these two variables, there were no other variable differences in either of the two groups. The junior high school students had a higher score in self-control than the university students, and the intrapersonal motivations of self-injury were more often chosen by the university group than by the junior high school students. Therefore, we combined the two groups to assess the relationships between the other psychological variables (**Supplementary Table S3**).

Regarding the frequency of NSSI behaviors (ISAS Section 1) and the motivations behind them (ISAS Section 2), there were significantly and positive related to depression, anxiety, and suicide ideation. To compare between the two factors (i.e., intrapersonal and interpersonal), factor scores were divided by the number of each subscale functions to make a mean score. There are five functions in the intrapersonal factor and eight in the interpersonal factor. The intrapersonal function was significantly correlated with depression and anxiety, while this result was not seen in the interpersonal function. Furthermore, the correlation between self-control and all other psychological variables were significantly negative, though all coefficients were not large.

## Discussion

The present study demonstrates that the Chinese version of the ISAS can be considered a suitable and reliable instrument for non-suicidal self-injury screening among young people in China. The behavioral and functional subscales of the ISAS both have adequate reliability and validity, and the two-factor structure of the NSSI functions have been identified and verified using both EFA and CFA, and our results are supported by similar findings from previous studies (26, 29). We also investigated the relationships between NSSI behaviors, NSSI functions, and other psychological variables such as depression, anxiety, suicidal ideation, and low self-control.

Firstly, the behavioral subscales all showed acceptable internal consistency, meaning that the Chinese version of the ISAS can be used to study lifetime frequencies of 12 types of NSSI behaviors in larger populations. The statistical properties of the Chinese version of the ISAS behavioral sections were found to be comparable to those reported in the original study, as well as in the Korean and Spanish studies (24, 26, 29). For instance, regarding internal consistency, each self-injurious behavior pattern was significantly correlated with the total frequency of NSSI as measured by the Chinese version of the ISAS. However, in contrast to other NSSI behaviors such as cutting, biting, carving, burning, and interfering with wound healing (e.g., picking scabs), the behavior of swallowing dangerous substances demonstrated weaker correlations with the total frequency of NSSI and was the least frequently endorsed in both participant groups in our study. This finding is consistent with previous research research (23, 25, 26, 30), indicating that this type of NSSI behavior may no longer be applicable to the current circumstances or requires further consideration.

Secondly, concerning the functional subscale of the ISAS, our analysis confirmed that it exhibits adequate internal consistency and validity. These findings are consistent with those reported in the original study as well as in the Persian, Spanish, and Korean adaptations (23, 24, 26, 29). Our results show a two-factor structure of the NSSI functions, which is in accordance with previous global research (50). There are two details that warrant attention. First, the function of “self-care” exhibited high loadings on both Factor 1 and Factor 2, which deviates from the classical two-factor model. However, previous studies (29, 33) have also found that “self-care” does not significantly differ between interpersonal factors and personal factors, or even exhibits higher factor loadings on the intrapersonal factor. Similarly, in the current study, “marking distress” had loadings below 0.6 in both groups, with only slight differences between the interpersonal and intrapersonal factors. Although this finding deviates from the original factor structure of the ISAS, it is also observed in other studies that have examined the factor structure of this measure (51). The discrepancies observed in the structural representation of “marking distress” and “self-care” in this study compared to other studies may be attributable to the limited sample size, potential cultural differences, or the specific selection of the participant population (primarily first-grade students). Overall, the structure of ISAS functions is nearly identical to that reported in previous studies (28, 52), with acceptable fit indices (NFI = 0.942, CFI = 0.974, IFI = 0.974, RMSEA = 0.068, SRMR = 0.043, CMIN/DF = 1.762), and can be considered as having a two-factor structure. Additionally, in terms of NSSI motivations, affective regulation emerged as the most frequently endorsed function, followed by self-punishment. Over 50% of participants in both groups endorsed affective regulation and self-punishment as contributing factors for their engagement in NSSI. These findings are consistent with prior research utilizing large samples to identify motives underlying NSSI behaviors (53). Such consistency underscores the global prevalence of affective regulation and self-punishment as salient motives among individuals who engage in non-suicidal self-injury. Future research should focus on these motives to elucidate their underlying mechanisms and broader implications.

The outcomes of the correlation analysis indicated that the self-reported NSSI functions, as assessed by the Chinese-version ISAS, exhibited a significant correlation with several clinically characteristics, including suicidal ideation, low self-control, anxiety, and depression. In other words, participants who exhibited more suicidal ideation, anxiety, depression, and lower self-control reported a greater number of functional motives for their self-harm behaviors. A similar correlation was observed between the frequency of NSSI behaviors and these clinical characteristics. This provides strong evidence for the expected relationship between psychopathology and NSSI. As previous studies have found, the prevalence of NSSI can be as high as 62.2% - 78.5% within a year among adolescents with mental disorders such as depression and bipolar disorder (54, 55). Anxiety levels show some ability to distinguish NSSI, although this distinction may lack unique significance in terms of the severity of NSSI (56). It is undeniable that depression and anxiety indeed interact with the motives and behaviors associated with NSSI. Considering the importance of self-control (57) for individuals in maintaining attention and focus as well as making and adhering to plans, which has been identified as a key focus of cognitive-behavioral therapy (CBT) interventions for individuals engaged in self-harm and suicide, it may be one of the valuable psychological resources. When vulnerable groups struggle to cope with internal and external environments, they may use self-control to alleviate impulsivity or seek external help, reducing the occurrence of NSSI behaviors and thus lowering the risk of suicide among adolescents in the future (5). Therefore, further research is needed to confirm the relationship between NSSI, suicidal ideation, and self-control, understand the development process of NSSI, such as longitudinal studies (58), and it is crucial to seek effective interventions to prevent the fatal consequences of self-harm behaviors.

These findings, along with the results of CFA and internal consistency, collectively support the internal construct validity of the scale.

Additionally, considering that we tested with two different participant groups, it is important to note that there were no statistically significant gender differences in the total ISAS scores for either the first or the second group. This indicates that gender is not a major factor influencing the frequency or motivation level of NSSI behaviors, at least in this study. This finding is both similar to and distinct from those from previous studies (59–62). However, we did find that there are some differences in trends that do exist between males and females in different group in terms of choice of specific NSSI methods and motivations. Thus, future research should focus on conventional variables such as gender and age, while also considering the potential influence of physiological factors including menarche (63) and brain development (64) on the occurrence of NSSI behavior.

We must acknowledge some limitations that exist in this study. First of all, given the shortcomings in sample size and sample construction, this study did not differentiate between the population samples included in the exploratory and confirmatory factor analyses of the scale, which may have tended to make the results favorable. Additionally, all samples included in the study were from schools, and the failure to explore school leavers who are commonly studied as being at greater risk for self-injury is a major weakness of this study in terms of scale generalizability. Secondly, though we built a model to fit the construct validity, this study is based on classic theory test (CTT), so we only tested a two-factor model and did not try to fit other possible models. Finally, all data collected were self-reported, which means that participants’ abilities or memories could have been affected by their environment. It should be emphasized that, according to the 2023 National 1‰ Population Sample Survey, the number of youths aged 15–24 in China was over 159 million at the end of 2023, accounting for 10.76% of the total population. The sample size and sample construction included in this study is clearly insufficient, which represents the most significant limitation of this paper. This issue affects the generalizability of our results. In future research, it is imperative to be more cautious about the composition of the sample, choosing to focus on more homogeneous groups or employing complex statistical methods to control for potential confounding effects. Future studies should adopt more rigorous sampling methods, such as stratified random sampling across different schools and socioeconomic backgrounds, to ensure greater representativeness of the sample. Additionally, future research should pay more attention to specific populations, utilize larger sample sizes, attempt item response theory (IRT), and further investigate the relationships between self-harm functions and other psychological characteristics in the context of Chinese culture.

## Conclusion

In conclusion, this study demonstrates that the Chinese version of the ISAS is a reliable and effective measure of NSSI frequency and function among Chinese adolescents, with its functional scales being well-explained by a two-factor structure of intrapersonal and interpersonal functions, consistent with previous research findings (28, 52). By validating the consistency of relevant scales across different cultural backgrounds and exploring the possible behavioral, psychological, and motivational factors underlying NSSI, we hope that the ISAS can serve as a standardized screening tool that is applicable within Chinese cultural contexts to identify adolescents at high risk for NSSI. Schools and medical institutions can then implement targeted interventions based on the identified risk factors and motivations. Additionally, the findings on the relationship between NSSI and psychological factors, such as depression and anxiety, can provide valuable insights for developing personalized treatment plans that address the specific needs of adolescents engaging in NSSI behaviors. We believe that this study will provide new research perspectives and methodological tools for understanding the psychological characteristics, patterns, and motivations of self-injury behavior among Chinese adolescents, and will serve as a catalyst for future validation research in this area.

## Data Availability

The raw data supporting the conclusions of this article will be made available by the authors, without undue reservation.
