# Supplementary material for: Non-suicidal self-injury in adolescence: a validation of the Chinese version of the Inventory of Statements About Self-Injury in student populations
Source: Front Psychiatry. 2025 Feb 27;16:1510681. doi: 10.3389/fpsyt.2025.1510681 (PMC11903741; doi:10.3389/fpsyt.2025.1510681)

**Table S1a**

| ISAS behavior | Table S1a. Group 1: University students(N=35) | | | | |
| --- | --- | --- | --- | --- | --- |
|  | *M* (*SD*) | Sample percentage (%) | | Correlations with the overall score | Cronbach's alpha if item deleted |
| Cutting | 1.54(1.04) | | 25.7 | 0.837** | 0.711 |
| Severe scratching | 1.34(0.73) | | 22.9 | 0.616** | 0.75 |
| Biting | 1.43(0.74) | | 28.6 | 0.809** | 0.724 |
| Banging or hitting self | 1.89(0.96) | | 54.3 | 0.521** | 0.77 |
| Burning | 1.06(0.24) | | 5.7 | 0.308** | 0.776 |
| Interfering with wound healing (e.g., picking scabs) | 2.23(1.24) | | 57.1 | 0.541** | 0.787 |
| Carving | 1.49(0.85) | | 28.6 | 0.758** | 0.729 |
| Rubbing skin against rough surface | 1.23(0.55) | | 17.1 | 0.475** | 0.764 |
| Pinching | 1.40(0.70) | | 28.6 | 0.583** | 0.754 |
| Sticking self with needles | 1.06(0.34) | | 2.9 | 0.572** | 0.762 |
| Pulling hair | 1.29(0.67) | | 17.1 | 0.217 | 0.79 |
| Swallowing dangerous substances | 1.00(0.00) | | 0 | - | - |
| Other | 1.06(0.34) | | 2.9 | 0.179 | 0.781 |
| Cronbach's alpha Based on standardized items: 0.788  ** Correlation is significant at the 0.01 level (two-tailed).  * Correlation is significant at the 0.05 level (two-tailed). | | | | | |

**Table S1b**

| ISAS behavior | Table S1b. Group 2: Junior high school students(N=132) | | | |
| --- | --- | --- | --- | --- |
|  | *M* (*SD*) | Sample percentage (%) | Correlations with the overall score | Cronbach's alpha if item deleted |
| Cutting | 1.71(0.93) | 43.9 | 0.503** | 0.78 |
| Severe scratching | 1.46(0.84) | 27.3 | 0.718** | 0.756 |
| Biting | 1.55(0.87) | 33.3 | 0.664** | 0.762 |
| Banging or hitting self | 1.82(1.08) | 43.2 | 0.673** | 0.761 |
| Burning | 1.21(0.58) | 13.6 | 0.464** | 0.78 |
| Interfering with wound healing (e.g., picking scabs) | 2.12(1.33) | 49.2 | 0.594** | 0.782 |
| Carving | 1.70(1.04) | 38.6 | 0.541** | 0.778 |
| Rubbing skin against rough surface | 1.52(0.93) | 29.5 | 0.647** | 0.764 |
| Pinching | 1.67(1.05) | 34.1 | 0.528** | 0.78 |
| Sticking self with needles | 1.17(0.50) | 11.4 | 0.481** | 0.78 |
| Pulling hair | 1.52(1.02) | 26.5 | 0.574** | 0.774 |
| Swallowing dangerous substances | 1.04(0.19) | 3.8 | 0.258** | 0.792 |
| Other | 1.05(0.09) | 3.8 | 0.222* | 0.792 |
| Cronbach's alpha Based on standardized items: 0.8  ** Correlation is significant at the 0.01 level (two-tailed).  * Correlation is significant at the 0.05 level (two-tailed). | | | | |

**Table S2a**

| Table S2a. Reliability Statistics for the University Group | | | | | |
| --- | --- | --- | --- | --- | --- |
|  | *M* (*SD*) | Sample percentage (%) | | Correlations with the overall score | Cronbach's alpha if item deleted |
| Interpersonal boundaries | 0.63 (1.0) | | 40 | .809** | 0.903 |
| Self-care | 1.14 (1.35) | | 62.9 | .802** | 0.902 |
| Sensation seeking | 0.66 (1.0) | | 37.1 | .746** | 0.905 |
| Peer bonding | 0.51 (1.27) | | 20 | .755** | 0.904 |
| Interpersonal influence | 0.83 (1.25) | | 40 | .779** | 0.903 |
| Toughness | 0.71 (1.23) | | 34.3 | .823** | 0.901 |
| Revenge | 0.46 (1.25) | | 17.1 | .791** | 0.903 |
| Autonomy | 0.46 (1.15) | | 17.1 | .771** | 0.904 |
| Affect regulation | 2.29 (1.6) | | 82.9 | .380* | 0.924 |
| Self-punishment | 1.94 (1.63) | | 80 | .675** | 0.909 |
| Anti-dissociation | 1.43 (1.31) | | 71.4 | .648** | 0.909 |
| Anti-suicide | 1.09 (1.58) | | 45.7 | .627** | 0.912 |
| Marking distress | 1.26 (1.69) | | 51.4 | .753** | 0.905 |
| Cronbach's alpha based on standardized items: 0.923. | | | | | |
| ** Correlation is significant at the 0.01 level (two-tailed). | | | | | |
| * Correlation is significant at the 0.05 level (two-tailed). | | | | | |

**Table S2b**

| Table S2b. Reliability Statistics for the Junior High School Group | | | | |
| --- | --- | --- | --- | --- |
|  | *M* (*SD*) | Sample percentage (%) | Correlations with the overall score | Cronbach's alpha if item deleted |
| Interpersonal boundaries | 0.40 (0.85) | 26.5 | .746** | 0.912 |
| Selfcare | 0.67 (1.07) | 38.6 | .813** | 0.908 |
| Sensation seeking | 0.33 (0.81) | 18.9 | .739** | 0.912 |
| Peer bonding | 0.42 (1.03) | 22 | .731** | 0.912 |
| Interpersonal influence | 0.41 (0.87) | 23.5 | .744** | 0.912 |
| Toughness | 0.56 (1.08) | 28 | .760** | 0.91 |
| Revenge | 0.17 (0.59) | 10.4 | .632** | 0.917 |
| Autonomy | 0.54 (1.02) | 28 | .714** | 0.913 |
| Affect regulation | 1.73 (1.54) | 70.5 | .706** | 0.917 |
| Self-punishment | 1.08 (1.35) | 52.3 | .764** | 0.911 |
| Anti-dissociation | 0.89 (1.23) | 45.5 | .832** | 0.907 |
| Anti-suicide | 1.02 (1.58) | 39.4 | .658** | 0.92 |
| Marking distress | 0.58 (1.01) | 32.6 | .694** | 0.913 |
| Cronbach's alpha based on standardized items: 0.930. | | | | |
| ** Correlation is significant at the 0.01 level (two-tailed). | | | | |
| * Correlation is significant at the 0.05 level (two-tailed). | | | | |

**Table S3**

*Correlations among ISAS and other Clinical Measures*

| Table S4. Correlations | | | | | | | | | | | | |
| --- | --- | --- | --- | --- | --- | --- | --- | --- | --- | --- | --- | --- |
|  | *M* | *SD* | NSSI-Section1 | NSSI-section2 | Inter-average | Intra-average | PHQ-9 | GAD-7 | SIOSS | BSCS | sex | age |
| NSSI Section 1 | 6.21 | 5.86 | 1 |  |  |  |  |  |  |  |  |  |
| NSSI Section 2 | 9.77 | 10.86 | .229** | 1 |  |  |  |  |  |  |  |  |
| Inter-average | 0.49 | 0.8 | .157* | .917** | 1 |  |  |  |  |  |  |  |
| Intra-average | 1.17 | 1.12 | .264** | .888** | .630** | 1 |  |  |  |  |  |  |
| PHQ-9 | 8.15 | 6.34 | .379** | .208** | 0.102 | .287** | 1 |  |  |  |  |  |
| GAD-7 | 5.55 | 5.43 | .360** | .223** | 0.139 | .273** | .807** | 1 |  |  |  |  |
| SIOSS | 8.4 | 5.72 | .374** | .322** | .246** | .342** | .657** | .661** | 1 |  |  |  |
| BSCS | 22.14 | 4.07 | -.191* | -.230** | -.187* | -.231** | -.255** | -.198* | -.225** | 1 |  |  |
| sex |  |  | -0.098 | 0.012 | 0.102 | -0.095 | -.261** | -.243** | -.267** | 0.12 | 1 |  |
| age | 14.46 | 3.13 | -0.108 | 0.142 | 0.104 | .156* | -0.008 | -0.053 | 0.011 | -.177* | -0.059 | 1 |
| ** Correlation is significant at the 0.01 level (two-tailed). | | | | | | | | | | | | |
| * Correlation is significant at the 0.05 level (two-tailed). | | | | | | | | | | | | |

**Figure S1**

*The modified model for the Chinese version of Inventory of Statements about Self-Injury (ISAS)*


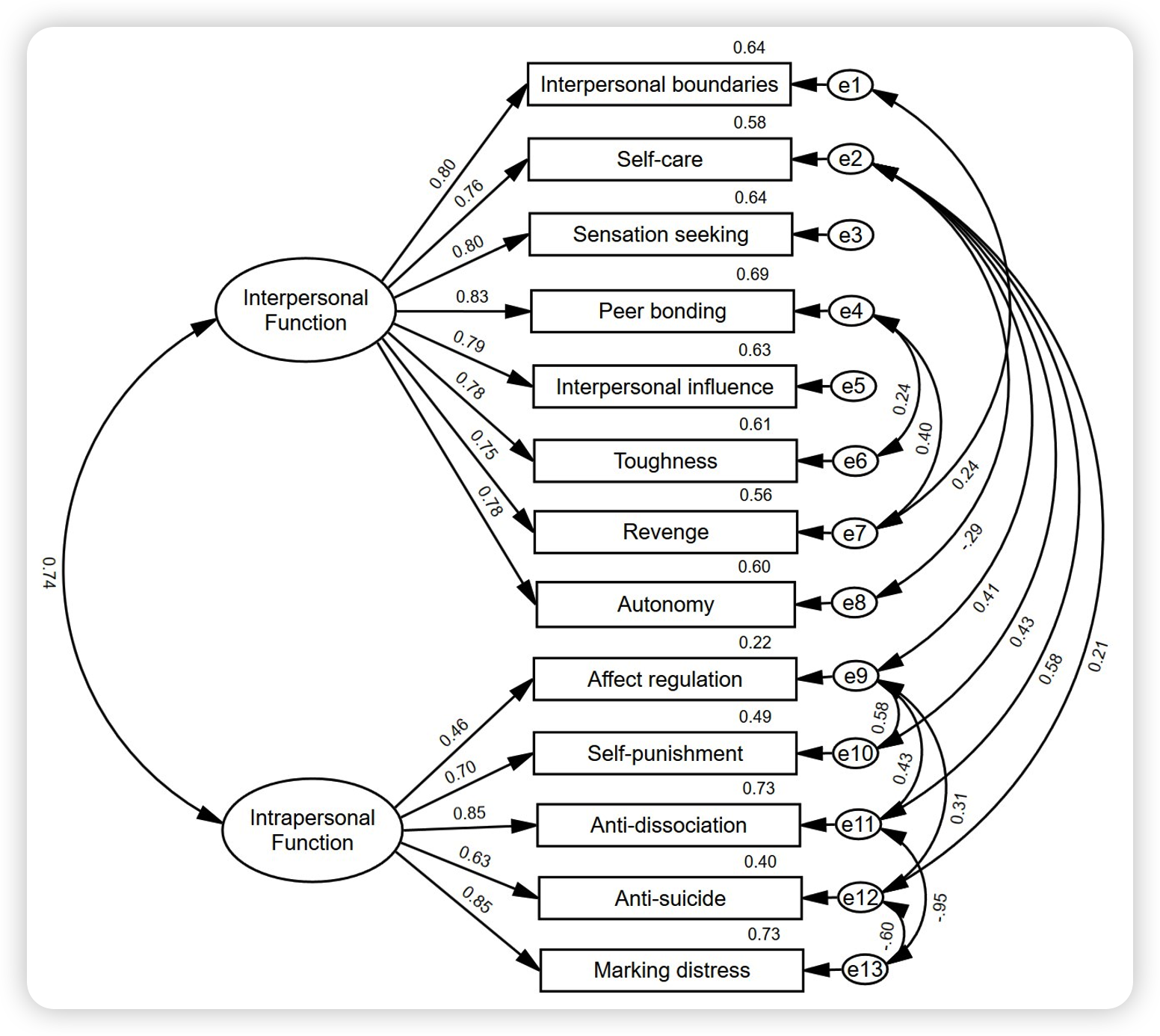

Supplement: Supplementary file 1 [file SupplementaryFile1.docx]
