# Supplementary material for: Non-suicidal self-injury in adolescence: a validation of the Chinese version of the Inventory of Statements About Self-Injury in student populations
Source: Front Psychiatry. 2025 Feb 27;16:1510681. doi: 10.3389/fpsyt.2025.1510681 (PMC11903741; doi:10.3389/fpsyt.2025.1510681)
Supplement: Supplementary file 2 [file SupplementaryFile2.docx]

Main abbreviation list

| ISAS | Inventory of Statements About Self-Injury |
| --- | --- |
| NSSI | Non-Suicidal Self-Injury |
| EFA | Exploratory Factor Analysis |
| CFA | Confirmatory Factor Analysis |
| PHQ-9 | Patient Health Questionnaire-9 |
| GAD-7 | Generalized Anxiety Disorder-7 |
| BSCS | Brief Self-Control Scale |
| SIOSS | Self-Rating Idea of Suicide Scale |
| M | Mean |
| SD | Standard Deviation |
| NFI | Normed Fit Index |
| CFI | Comparative Fit Index |
| IFI | Incremental Fit Index |
| CMIN/DF | Chi-square Minimum/Degree of Freedom |
| RMSEA | Root Mean Square Error of Approximation |
| SRMR | Standardized Root Mean Squared Residual |
| UniCo | Unidimensional Congruence |
| ECV | Explained Common Variance |
| MIREAL | Mean of Item Residual Absolute Loadings |
| PA | Parallel Analysis |
| FASM | Functional Assessment of Self-Mutilation |
| SIQ-TR | Self-Injury Questionnaire-Treatment Related |
| SITBI | Self-Injurious Thoughts and Behavior Interview |
| NSSI-AT | Non-Suicidal Self-Injury Assessment Tool |
| CTT | Classic Theory Test |
| IRT | Item Response Theory |
