# Supplementary material for: Non-suicidal self-injury in adolescence: a validation of the Chinese version of the Inventory of Statements About Self-Injury in student populations
Source: Front Psychiatry. 2025 Feb 27;16:1510681. doi: 10.3389/fpsyt.2025.1510681 (PMC11903741; doi:10.3389/fpsyt.2025.1510681)
Supplement: Supplementary file 3 [file SupplementaryFile3.docx]

STROBE Statement—checklist of items that should be included in reports of observational studies

|  | Item No. | Recommendation | | Relevant text from manuscript | |
| --- | --- | --- | --- | --- | --- |
| **Title and abstract** | 1 | (*a*) Indicate the study’s design with a commonly used term in the title or the abstract | | “Non-Suicidal Self-Injury in Adolescence: A Validation of the Chinese Version of the Inventory of Statements About Self-Injury in Student Populations” | |
|  |  | (*b*) Provide in the abstract an informative and balanced summary of what was done and what was found | | “This study validates the Chinese ISAS as a reliable NSSI measure, identifies a two-factor structure, and aims to inform targeted interventions and future research on self-injury behaviors among Chinese adolescents.” | |
| Introduction | | |  | |  |
| Background/rationale | 2 | Explain the scientific background and rationale for the investigation being reported | | “Nonsuicidal self-injury (NSSI) refers to a range of specific self-injurious behaviors (SIB) such as intentional injury of body tissue without subjective suicidal intent(1,2). ……This, in turn, will facilitate the development and implementation of more effective support and intervention strategies.” | |
| Objectives | 3 | State specific objectives, including any prespecified hypotheses | | “The current study had two goals: the first was to investigate the factor structure and internal consistency of the Chinese version of the Inventory of Statements about Self-Injury (ISAS), and the second was to evaluate a specific demographic’s behavior and their motivation for NSSI behaviors. It was hypothesized that the Chinese version of the ISAS would establish a well two-factor structure providing adequate psychometric features.” | |
| Methods | | |  | |  |
| Study design | 4 | Present key elements of study design early in the paper | | “To create the Chinese version of the ISAS we adopted a back-translation approach(42). The forward translation (from English to Chinese) was carried out by the first author (XT), who is a graduate student in psychiatry with a focus on research related to self-injurious behaviors. ……In the present study, only students who reported a history of NSSI were included in the subsequent statistical analyses.” | |
| Setting | 5 | Describe the setting, locations, and relevant dates, including periods of recruitment, exposure, follow-up, and data collection | | “The study cohort comprised Chinese adolescents aged 12 to 24 years, who were enrolled from two schools participating in distinct research projects during the period from October to December 2021. The majority of participants were from a secondary school in Huaiji, a county in Guangzhou. In total, 706 first year junior high school students completed the questionnaire online, among who 182 students reported NSSI history. The remaining participants were recruited from Guangzhou Sport University and completed the questionnaire on Redcap (a questionnaire distribution platform). In total, 464 completed questionnaires were received from the Sport University students, among who 49 students reported NSSI history.” | |
| Participants | 6 | (*a*) *Cohort study*—Give the eligibility criteria, and the sources and methods of selection of participants. Describe methods of follow-up  *Case-control study*—Give the eligibility criteria, and the sources and methods of case ascertainment and control selection. Give the rationale for the choice of cases and controls  *Cross-sectional study*—Give the eligibility criteria, and the sources and methods of selection of participants | | “Participants who reported NSSI history were excluded if their responses were inconsistent or obviously irrational. 14 university and 44 junior high school students were excluded because they had not done any self-harm behavior but reported at least one way of NSSI behavior. Six junior high school students were excluded because their responses were irrational (i.e. times of self-harm behavior >1000 or irrelevant answers with NSSI history).  After excluded these participants, a total of 1,106 questionnaires were deemed eligible (450 university students and 656 adolescents from the junior high school student group). A total of 167 students, consisting of 132 high school students and 35 university students, reported engaging in at least one incident of NSSI during their lifetime. This final sample of 167 NSSI students was used in the validation study. | |
|  |  | (*b*) *Cohort study*—For matched studies, give matching criteria and number of exposed and unexposed  *Case-control study*—For matched studies, give matching criteria and the number of controls per case | | NA | |
| Variables | 7 | Clearly define all outcomes, exposures, predictors, potential confounders, and effect modifiers. Give diagnostic criteria, if applicable | | “Inventory of Statements about Self-Injury (ISAS). The scale is made up of two parts. The first part of the ISAS assesses the lifetime frequency of 12 “intentional” (i.e., on purpose) and “non-suicidal” self-injury behavior types and one blank which can be filled out by participants in the event that they had engaged in another self-harm behavior type not already mentioned……. Generally, a total score of 12 or more is considered to be presence of suicidal ideation.” | |
| Data sources/ measurement | 8* | For each variable of interest, give sources of data and details of methods of assessment (measurement). Describe comparability of assessment methods if there is more than one group | | “Data were analyzed using SPSS 26 for Windows. Descriptive analyses (mean [M], standard deviation [SD], and frequencies) were used for the sample description and quantitative items of the ISAS……. Bivariate correlation analyses were used to examine whether the ISAS and its subscales correlated with other variables in the expected directions, which helped evaluate the measure’s criterion related validity.” | |
| Bias | 9 | Describe any efforts to address potential sources of bias | | “To create the Chinese version of the ISAS we adopted a back-translation approach(42). The forward translation (from English to Chinese) was carried out by the first author (XT), who is a graduate student in psychiatry with a focus on research related to self-injurious behaviors. This ensured a profound comprehension of the original scale's content and context. Subsequently, the backward translation (from Chinese to English) was performed by another graduate student in psychiatry (HH), who is proficient in both English and Chinese and has a solid research background in mental health. Both of these steps were supervised by the senior author (KL), who has extensive experience in scale development and validation. Thereafter, a panel of experts, including clinicians and researchers with expertise in psychiatry and psychometrics (such as WL, RZ, RW, etc.), compared and reconciled the translations to ensure the accuracy and cultural appropriateness of the translated items.” | |
| Study size | 10 | Explain how the study size was arrived at | | NA | |

Continued on next page

| Quantitative variables | 11 | Explain how quantitative variables were handled in the analyses. If applicable, describe which groupings were chosen and why | | “Data were analyzed using SPSS 26 for Windows. Descriptive analyses (mean [M], standard deviation [SD], and frequencies) were used for the sample description and quantitative items of the ISAS.” | |
| --- | --- | --- | --- | --- | --- |
| Statistical methods | 12 | (*a*) Describe all statistical methods, including those used to control for confounding | | “Data were analyzed using SPSS 26 for Windows. Descriptive analyses (mean [M], standard deviation [SD], and frequencies) were used for the sample description and quantitative items of the ISAS……. Bivariate correlation analyses were used to examine whether the ISAS and its subscales correlated with other variables in the expected directions, which helped evaluate the measure’s criterion related validity.” | |
|  |  | (*b*) Describe any methods used to examine subgroups and interactions | | “To examine whether the ISAS CFA model differs between genders and age groups, we conducted a multi-group simultaneous analysis. A critical ratio with an absolute value exceeding 1.96 was considered to indicate a significant difference, when the significance level was set at 0.05.  Bivariate correlation analyses were used to examine whether the ISAS and its subscales correlated with other variables in the expected directions, which helped evaluate the measure’s criterion related validity. In the present study, only students who reported a history of NSSI were included in the subsequent statistical analyses.” | |
|  |  | (*c*) Explain how missing data were addressed | | “The age data for some university students were missing(N=4), but these gaps were filled using the mean substitution method.”” | |
|  |  | (*d*) *Cohort study*—If applicable, explain how loss to follow-up was addressed  *Case-control study*—If applicable, explain how matching of cases and controls was addressed  *Cross-sectional study*—If applicable, describe analytical methods taking account of sampling strategy | | NA | |
|  |  | (*e*) Describe any sensitivity analyses | | NA | |
| Results | | |  | |  |
| Participants | 13* | (a) Report numbers of individuals at each stage of study—eg numbers potentially eligible, examined for eligibility, confirmed eligible, included in the study, completing follow-up, and analysed | | NA | |
|  |  | (b) Give reasons for non-participation at each stage | | NA | |
|  |  | (c) Consider use of a flow diagram | | NA | |
| Descriptive data | 14* | (a) Give characteristics of study participants (eg demographic, clinical, social) and information on exposures and potential confounders | | NA | |
|  |  | (b) Indicate number of participants with missing data for each variable of interest | | NA | |
|  |  | (c) *Cohort study*—Summarise follow-up time (eg, average and total amount) | | NA | |
| Outcome data | 15* | *Cohort study*—Report numbers of outcome events or summary measures over time | | NA | |
|  |  | *Case-control study—*Report numbers in each exposure category, or summary measures of exposure | | NA | |
|  |  | *Cross-sectional study—*Report numbers of outcome events or summary measures | | NA | |
| Main results | 16 | (*a*) Give unadjusted estimates and, if applicable, confounder-adjusted estimates and their precision (eg, 95% confidence interval). Make clear which confounders were adjusted for and why they were included | | “Of the two different groups of NSSI participants, university students account for 21.0%. 48.6% of the university students were male(N=, and 42.4% of the junior high school students were female……. The correlation value (0.74) between the two latent variables indicated that these two can be interpreted by one higher order latent variable, which can be considered to be a total ISAS function. The modified model for the ISAS is shown in Figure S1.” | |
|  |  | (*b*) Report category boundaries when continuous variables were categorized | | NA | |
|  |  | (*c*) If relevant, consider translating estimates of relative risk into absolute risk for a meaningful time period | | NA | |

Continued on next page

| Other analyses | 17 | Report other analyses done—eg analyses of subgroups and interactions, and sensitivity analyses | | “To examine the potential effects of respondents' gender and age on W1-11 in Figure 3, multi-group simultaneous analyses were conducted for each function subscale of ISAS. Most parameters did not show significant differences in terms of gender and age……. Furthermore, the correlation between self-control and all other psychological variables were significantly negative, though all coefficients were not large.” | |
| --- | --- | --- | --- | --- | --- |
| Discussion | | |  | |  |
| Key results | 18 | Summarise key results with reference to study objectives | | “The present study demonstrates that the Chinese version of the ISAS can be considered a suitable and reliable instrument for non-suicidal self-injury screening among young people in China. The behavioral and functional subscales of the ISAS both have adequate reliability and validity, and the two-factor structure of the NSSI functions have been identified and verified using both EFA and CFA, and our results are supported by similar findings from previous studies(26,29). We also investigated the relationships between NSSI behaviors, NSSI functions, and other psychological variables such as depression, anxiety, suicidal ideation, and low self-control.” | |
| Limitations | 19 | Discuss limitations of the study, taking into account sources of potential bias or imprecision. Discuss both direction and magnitude of any potential bias | | “We must acknowledge some limitations that exist in this study. First of all, given the shortcomings in sample size and sample construction, this study did not differentiate between the population samples included in the exploratory and confirmatory factor analyses of the scale, which may have tended to make the results favorable. Additionally, all samples included in the study were from schools, and the failure to explore school leavers who are commonly studied as being at greater risk for self-injury is a major weakness of this study in terms of scale generalizability. Secondly, though we built a model to fit the construct validity, this study is based on classic theory test (CTT), so we only tested a two-factor model and did not try to fit other possible models. Finally, all data collected were self-reported, which means that participants’ abilities or memories could have been affected by their environment. It should be emphasized that, according to the 2023 National 1‰ Population Sample Survey, the number of youths aged 15–24 in China was over 159 million at the end of 2023, accounting for 10.76% of the total population. The sample size and sample construction included in this study is clearly insufficient, which represents the most significant limitation of this paper. This issue affects the generalizability of our results.” | |
| Interpretation | 20 | Give a cautious overall interpretation of results considering objectives, limitations, multiplicity of analyses, results from similar studies, and other relevant evidence | | “Firstly, the behavioral subscales all showed acceptable internal consistency, meaning that the Chinese version of the ISAS can be used to study lifetime frequencies of 12 types of NSSI behaviors in larger populations……. Thus, future research should focus on conventional variables such as gender and age, while also considering the potential influence of physiological factors including menarche(63) and brain development(64) on the occurrence of NSSI behavior. ” | |
| Generalisability | 21 | Discuss the generalisability (external validity) of the study results | | “In conclusion, this study demonstrates that the Chinese version of the ISAS is a reliable and effective measure of NSSI frequency and function among Chinese adolescents, with its functional scales being well-explained by a two-factor structure of intrapersonal and interpersonal functions, consistent with previous research findings(28,52). By validating the consistency of relevant scales across different cultural backgrounds and exploring the possible behavioral, psychological, and motivational factors underlying NSSI, we hope that the ISAS can serve as a standardized screening tool that is applicable within Chinese cultural contexts to identify adolescents at high risk for NSSI. Schools and medical institutions can then implement targeted interventions based on the identified risk factors and motivations. Additionally, the findings on the relationship between NSSI and psychological factors, such as depression and anxiety, can provide valuable insights for developing personalized treatment plans that address the specific needs of adolescents engaging in NSSI behaviors. We believe that this study will provide new research perspectives and methodological tools for understanding the psychological characteristics, patterns, and motivations of self-injury behavior among Chinese adolescents, and will serve as a catalyst for future validation research in this area.” | |
| Other information | |  |  |  |  |
| Funding | 22 | Give the source of funding and the role of the funders for the present study and, if applicable, for the original study on which the present article is based | | “This work was supported by Science and Technology Program of Guangzhou, China (No. 202007030012), the National Natural Science Foundation of China (NSFC: 82171531), Guangzhou Municipal Key Discipline in Medicine (2021-2023), Guangzhou High-level Clinical Key Specialty, Guangzhou Research-oriented Hospital, and Guangzhou Health science and Technology general guidance project (No. 20221A011052).” | |

*Give information separately for cases and controls in case-control studies and, if applicable, for exposed and unexposed groups in cohort and cross-sectional studies.

**Note:** An Explanation and Elaboration article discusses each checklist item and gives methodological background and published examples of transparent reporting. The STROBE checklist is best used in conjunction with this article (freely available on the Web sites of PLoS Medicine at http://www.plosmedicine.org/, Annals of Internal Medicine at http://www.annals.org/, and Epidemiology at http://www.epidem.com/). Information on the STROBE Initiative is available at www.strobe-statement.org.
